# Historia de la tos ferina en Colombia: muertes infantiles endémicas, brotes y epidemias

**DOI:** 10.7705/biomedica.8075

**Published:** 2026-08-20

**Authors:** Álvaro J. Idrovo, Claudia Amaya-Castellanos, Silvana Zapata-Bedoya

**Affiliations:** 1 Departamento de Salud Pública, Escuela de Medicina, Universidad Industrial de Santander, Bucaramanga, Colombia Universidad Industrial de Santander Departamento de Salud Pública Escuela de Medicina Universidad Industrial de Santander Bucaramanga Colombia; 2 Organización Panamericana de la Salud, Medellín, Colombia Organización Panamericana de la Salud Medellín Colombia

**Keywords:** tos ferina/epidemiología, historia, epidemias, enfermedades endémicas, salud infantil, Whooping cough/epidemiology, history, epidemics, endemic diseases, child health

## Abstract

La frecuencia de la tos ferina fue controlada en varios países con la vacunación, aunque ha reemergido en las últimas décadas. Poco se sabe de la historia de esta enfermedad en Colombia; este trabajo resume la historia de la tos ferina en el país desde un enfoque poblacional.

Las evidencias sugieren que la tos ferina apareció hacia finales del siglo XV en Persia, y desde esa época ha sido endémica, especialmente entre los menores de edad. Al territorio colombiano debió llegar durante el siglo XVIII o a inicios del XIX.

En Colombia, se han presentado importantes epidemias, en especial antes de que existiera la vacuna contra *Bordetella pertussis.* Las epidemias más importantes tuvieron lugar en Bogotá (1814) y en Cajicá (1922).

La vacunación fue muy efectiva en la segunda mitad del siglo XX, aunque hubo brotes entre las poblaciones no vacunadas de las zonas rurales y entre los indígenas de diversos lugares del país. Actualmente, la tos ferina es una amenaza para Colombia por los casos traídos del exterior o en personas sin vacunar.

## Abstract (en)

The occurrence of whooping cough was controlled with vaccination in several countries, but it has re-emerged in recent decades. Little is known about the history of the disease in Colombia. This paper summarizes the history of whooping cough in Colombia, viewed from a population perspective.

Evidence suggests that whooping cough appeared towards the end of the 15^th^. century in Persia, and since then it has been mostly endemic among children. It must have arrived in Colombia during the 18^th^. or early 19^th^. century. In Colombia, major epidemics have occurred, especially before the availability of the *Bordetella pertussis* vaccine. The most significant epidemics occurred in Bogotá (1814) and Cajicá (1922).

Vaccination was very effective in the second half of the 20^th^. century, although there were outbreaks among unvaccinated populations in rural and indigenous areas in various parts of the country. Whooping cough is currently a threat to Colombia due to cases brought in from abroad or unvaccinated individuals.

En Latinoamérica, el interés por la historia de las enfermedades como campo de estudio, se ha incrementado en las últimas dos décadas ^1^. Cuando esta disciplina se ocupa de la presentación de las enfermedades en las poblaciones y no en los individuos, se vuelve tema de la historia de la salud pública ^2^.

Precisamente, en las poblaciones es donde la presencia de las enfermedades puede describirse de manera simple como endemia o epidemia. En el primer caso, su frecuencia se mantiene dentro de unos valores considerados habituales a lo largo del tiempo ^3^, lo que permite la inclusión de comportamientos cíclicos en los que aumenta y disminuye de forma repetida. En el caso de las epidemias, los casos aumentan por encima de la frecuencia habitual, y pueden tornarse en crisis sanitarias con diferentes niveles de afectación social; además, suelen existir factores determinantes sociales o ambientales relacionados que actúan como desencadenantes ^4^.

En este contexto, el presente trabajo aborda la historia de la tos ferina en Colombia desde un enfoque poblacional; por ello, busca identificar periodos y lugares en los que la enfermedad se comportó como endemia o como epidemia, así como los posibles factores determinantes asociados. El tema resulta de especial interés dado que la tos ferina, pese a la vacunación, es endémica en varias regiones del país y a que ha resurgido en diversos lugares del mundo donde se pensaba que estaba controlada ^5^.

En Colombia, el Sistema de Vigilancia en Salud Pública (Sivigila) ha evidenciado un incremento del número de casos de tos ferina en los últimos años, con una incidencia de 0,12 casos por cada 100 000 habitantes en el 2024, afectando principalmente a los menores de edad ^6^. Esto, sin duda, amerita toda la atención, por lo que conocer la historia de la enfermedad en el país resulta relevante.

Es importante señalar que la tos ferina ocurre por la infección por *Bordetella pertussis,* y que un individuo puede volverse a infectar después de un tiempo, por lo cual es necesario el refuerzo vacunal ^7^. Desde los sistemas dinámicos, la tos ferina es una infección con un patrón cíclico dominante cuya periodicidad varía según la cobertura de la vacunación. En las poblaciones con vacunación, los ciclos epidémicos pueden variar entre los 3,5 y los 5 años ^8^. Los estudios de las poblaciones chilenas han demostrado que, en los periodos sin vacunación, este lapso puede acortarse a 2 o 3 años, dependiendo en gran medida de la natalidad que, al disminuir, ocasiona que los ciclos de infección se distancien en el tiempo ^9^.

Además, un efecto esperable es que, con amplias coberturas de vacunación y buena vigilancia en salud pública, se detecten más fácilmente los brotes de infección.

## Origen y primera pandemia

La tos ferina (del italiano *tosse ferina* ) también es conocida como coqueluche, *whooping cough, chyncough, chincofe, kink, kinkhost, kichhosta, kirkhosta, kighoste, keichhusten, kwinthoest, coclude, tussis* o *clongosa* en los libros antiguos de medicina ^10^. Estas múltiples denominaciones hacen difícil rastrear la enfermedad en los tiempos pasados. Dada la tos excesiva que caracteriza la enfermedad, el nombre de *pertussis* ^11^ y el de tos ferina son las formas de denominación más comunes en la actualidad.

El caso confirmado de infección más antiguo se presentó en un anciano perteneciente a la élite social de Siberia, que vivió a finales del siglo XVII o comienzos del XVIII; el análisis molecular del tejido pulmonar confirmó la presencia de *B. pertussis* ^12^. Dado que no existen descripciones de la enfermedad en los escritos antiguos de la medicina griega, romana o árabe, varios autores han propiciado la controversia de si la tos ferina era una enfermedad «nueva» en la Europa del siglo XV o si no lo era ^13^.

Son muy pocas las descripciones de cuadros clínicos sugestivos de tos ferina antes de esta fecha; una de ellas es el reporte de sanación milagrosa de un niño con *tussis vehementiore,* o tos violenta *(kinkehost),* atribuida a san Godric de Finchale hacia 1190. Otro caso está descrito en el *Miracle book of St. John's Cathedral,* en el sur de los Países Bajos, como un caso de sanación milagrosa de un niño con *kychoest* en 1383, relacionado con los rezos a una estatua de Santa María ^14^.

Los recientes estudios de genómica han permitido comprender mejor el origen de la enfermedad. Las evidencias señalan que las bacterias del género *Bordetella* tienen como principal hábitat el suelo y que, de allí, surgió *B. bronchiseptica,* antecesora de la especie que infecta a los humanos ^15^.

Con base en la diversidad genética y en las relaciones evolutivas de las especies de *Bordetella,* se ha sugerido que *B. pertussis* tuvo un origen zoonótico, desarrollándose de *B. bronchisepteptica* que actualmente se puede encontrar en perros, conejos, ovejas, cerdos y humanos. La relación entre *B. pertussis* y los humanos, al parecer, se dio desde el período neolítico ^16^.

Un estudio histórico reciente reportó que en los *Khulāsāt al-tajārib ***,**** manuscritos médicos islámicos antiguos, se describen tres epidemias de tos ferina en Persia, que pueden considerarse las primeras descritas en todo el mundo. Entre 1484 y 1495 en la ciudad de Herat, actual Afganistán, ocurrieron dos epidemias que afectaron menores de edad y adultos; el afectar a los adultos se considera como la evidencia de que era una enfermedad emergente. La otra epidemia ocurrió en 1501 en la ciudad de Rey, muy cerca de Teherán -la capital actual de Irán-, y de allí se puede relacionar con las epidemias de Francia del siglo XVI ^17^.

En resumen, estos hallazgos sugieren que *B. pertussis* ha permanecido con los seres humanos desde hace 2,5 millones de años en casos aislados; después de las epidemias de Persia llegó a Europa y de allí a todo el mundo. Es probable que la ruta de la seda haya servido como recorrido de la propagación de la enfermedad.

## Del Viejo Mundo al Nuevo Mundo

Historiadores como Robert Weston señalan que no hay evidencia de epidemias de tos ferina antes del siglo XV ^10^. Sin embargo, la falta de claridad de los cuadros clínicos de la enfermedad impide que los estudiosos se pongan de acuerdo en cuáles epidemias fueron de tos ferina. Por ejemplo, Adalbert Friedrich Marcus, en su *Traité de la coqueluche,* publicado en 1821, informa 37 epidemias en Europa, las Indias orientales y en el centro, sur y oriente de América; en su listado, la epidemia más antigua ocurrió en 1414 en Francia, donde -señalaba- también hubo epidemias en 1510, 1558 y 1580 ^18^. Allí no se señala la epidemia de 1578 descrita por Guillaume de Baillou como de coqueluche en los dos libros publicados ^19^; no debe olvidarse que de Baillou es considerado uno de los primeros epidemiólogos modernos ^20^. Complicando más el asunto, Achille Chéreau señala que las epidemias de 1414 y 1580 no eran de tos ferina, sino de influenza ^21^.

Por otra parte, se ha propuesto que el agente causal del «gran catharro» que afectó a Madrid, Barcelona y muchos poblados españoles en 1580 ^22^, fue el de la tos ferina ^23^. Pese a las discrepancias en la identificación del agente causal de varias epidemias, hay consenso en que la mayoría de las epidemias de tos ferina en Europa hasta antes de 1700 ocurrieron en Francia, sumándose España e Italia en la epidemia de 1580 ^10^. Al parecer, durante los siglos XVIII y XIX, la tos ferina era endémica en varios países europeos ^11^. Inglaterra y Gales tienen registros que muestran disminución de la mortalidad por tos ferina en la segunda mitad del siglo XIX ^24^.

Para la historiadora mexicana Elsa Malvido, la primera epidemia de tos ferina en América ocurrió en México en 1564, luego de que los indígenas empezaron a ser congregados en los nuevos asentamientos organizados por los españoles; según indica, entre los indígenas, la enfermedad fue conocida como *tlatlacistli* ^25^. Debe señalarse que su temprana presentación puede ocasionar cuestionamientos sobre si realmente fue tos ferina.

Por su parte, el médico historiador Francisco Guerra señala que en 1659 aparecieron casos de tos ferina en Boston y en sus alrededores ^26^. En los estados de Nueva Inglaterra y en Carolina del Sur en los Estados Unidos de América, hubo epidemias de coqueluche en 1659 y en 1738, respectivamente; luego, hacia finales del siglo XVIII, la epidemia se expandió a todo el país ^26^.

Ya entrado el siglo XVI se empezó a tener claridad de la gravedad de la enfermedad y se señaló como grupo de riesgo a los menores de edad. Una descripción clínica muy conocida de esta «tos del mentón» se debe a Thomas Willis quien, en 1674, mencionó la presencia de tos frecuente muy intensa, respiración dolorosa, con interrupciones durante la inspiración o la espiración, simulando un estrangulamiento al toser ^27^.

En 1693, ocurrió una gran epidemia de «quinta» en Roma y París, y otra en Inglaterra en 1724. Entre 1732 y 1733, la tos ferina fue muy frecuente en toda Europa y, luego, se expandió a Jamaica, Perú y México, transmitida por los marineros ingleses ^28^. De hecho, en 1738, ocurrió una epidemia en Carolina del Sur con gran mortalidad ^26^.

La gran letalidad de la tos ferina fue muy evidente después de que los registros suecos reportaron 2712 muertes infantiles en promedio por año (~151 por 100 000 habitantes) entre 1749 y 1764 ^29^. Posteriormente, en 1794, después del verano, hubo una epidemia en Nueva York ^26^.

Entre las epidemias de tos ferina del siglo XIX, recopiladas por Francisco Guerra ^26^, sobresalen:

- las de Costa Rica: la de 1801, que dejó muchos muertos; la de 1861, que dejó «los más tristes recuerdos», y la de 1891 a 1893, que ocasionó cerca de 8000 muertes de menores de edad;
- las epidemias de Guatemala que, en 1804, 1866 y 1869, afectaron un número no determinado de menores de edad de la capital y, en 1876, ocasionó 4 muertes entre 82 enfermos;
- la de «toses convulsivas» de Lima (Perú) en 1808 ^30^;
- la de Honduras en 1867, que ocasionó 60 muertes en un tiempo corto, y
- la de Quito (Ecuador) en 1890 ^26^.

En Inglaterra y Gales, hubo dos epidemias de tos convulsiva con elevado número de fallecidos (63 381 casos) en los periodos de 1832 a 1842 y de 1847 a 1849 ^28^. Otras ciudades europeas tuvieron incrementos de la mortalidad por tos ferina, aunque en una escala menor ^10^.

En Chile, con mejores registros, hubo epidemias en La Serena en 1851, en Valdivia en 1853, y en Magallanes en 1890; entre 1892 y 1895, hubo 8181 fallecidos por coqueluche ^31^^,^^32^. Por este mismo tiempo, en Hawái también se describió tos ferina epidémica en 1848-1849, acompañada de sarampión, disentería e influenza ^33^.

En el estado de Paraná (Brasil), se describieron varias epidemias de tos ferina asociadas con precarias condiciones sociales en: Paranaguá (1855 a 1858), Morretes (1857, 1863 y 1886), Curitiba (1857, 1864 a 1865 y 1885 a 1888), Rio Negro (1873), Ponta Grossa (1878), Antonina (1886) y São José (1886) ^34^^,^^35^.

En Francia, durante el siglo XIX y comienzos del XX, ocurrieron frecuentes epidemias que afectaron a los menores de edad más pequeños; se conocen las epidemias de 1867-1874, 1880-1890, 1895, 1896-1908 y 1910-1914 ^36^.

En 1910, el médico Francisco Labrador González describió una epidemia de coqueluche en Villardefrades (España), siendo una de las mejor estudiadas de aquel periodo; duró del 11 de enero al 19 de abril, y afectó 67 individuos, la mayoría con edades entre uno y cuatro años (58,21 %), con una mortalidad del 1,5 % ^37^.

A comienzos del siglo XX, en Europa, la tos ferina seguía siendo una enfermedad endémica de comportamiento cíclico, en algunos casos, coincidiendo con los periodos estacionales 38. En 1918, en Costa Rica, ocurrió una epidemia de tos ferina ^39^, evidencia adicional de la enfermedad en América.

## Agente causal, vacuna y reemergencia

En 1906, Jules Bordet y Octave Gengou identificaron a *B. pertussis* como agente causal de la tos ferina ^40^, iniciándose un cambio en la comprensión de la enfermedad. Esto permitió que se iniciaran trabajos para producir vacunas, lo cual se logró en 1914 con la primera vacuna de células enteras con gran efectividad ^41^; posteriormente, se desarrollaron varias vacunas con muy heterogénea efectividad. Luego surgieron las vacunas de toxoide diftérico y tetánico, y la de tos ferina acelular, lo que permitió que, en 1948, fueran combinadas en la llamada vacuna DPT ^42^.

En la Asamblea Mundial de la Salud de 1974, se aprobó el Plan Ampliado de Inmunización (PAI) y sus metas fueron respaldadas mediante la Resolución CD 25.27 del Consejo Directivo de la Organización Panamericana de la Salud (OPS) y la Organización Mundial de la Salud (OMS) en septiembre de 1977, como medida para intensificar la lucha contra las enfermedades inmunoprevenibles que afectaban con mayor frecuencia a la niñez del mundo: tuberculosis, sarampión, difteria, tétanos, poliomielitis y tos ferina, poniéndose como meta llegar al 80 % de la vacunación infantil ^43^. Para ello, la vacuna DPT resultó ser un elemento básico de la estrategia.

La vacunación fue exitosa en los países de altos ingresos, por lo que el reto era que fuese un logro global; por tanto, en 1977, la Asamblea Mundial de Salud adoptó el objetivo de lograr la inmunización infantil universal para 1990. Para cumplir este fin, se buscó mejorar los sistemas de vigilancia y celebrar días de vacunación nacional. En 1982, el PAI se consolidó como componente esencial de la atención primaria en salud. A estas iniciativas se sumaron la Fundación Rockefeller, el Banco Mundial, la UNICEF y otras organizaciones ^44^.

En estos mismos años, se discutía la frecuencia de reacciones adversas asociadas con las vacunas, lo cual generó preocupación por su seguridad y el estancamiento de la cruzada para su control ^41^^,^^42^. Por ello, se realizaron estudios clínicos durante las décadas de los 80 y los 90 que permitieron hacer análisis críticos, a partir de los cuales se identificó que la seguridad había mejorado con el cambio de las técnicas de producción y que la poca efectividad se debía, en gran medida, a que los adultos servían de reservorio de la infección, por lo cual, si estos no eran vacunados, sería imposible la prevención de los brotes epidémicos ^45^.

Pese a las amplias coberturas de vacunación, en las últimas tres décadas se ha observado un resurgimiento global de la tos ferina esporádica y epidémica, aunque no en las magnitudes de los tiempos anteriores a la vacuna.

A pesar de la disponibilidad generalizada de vacunas y de los logros alcanzados en términos de cobertura, la tos ferina ha persistido históricamente como una de las principales causas de mortalidad de la población infantil en el mundo. Según las proyecciones recientes, para el 2014, se estimó que la enfermedad hab**í**a ocasionado cerca de 24 millones de casos y de 160 700 defunciones de niños menores de cinco años a nivel global. Estas aproximaciones ponen de relieve la importancia epidemiológica de la tos ferina como factor determinante de la mortalidad infantil; no obstante, deben interpretarse con cautela debido a la carencia de datos consolidados de vigilancia y a las limitaciones diagnósticas que históricamente han condicionado la notificación precisa de la enfermedad ^46^.

Los avances en el conocimiento de las epidemias de tos ferina diferencian claramente los periodos anteriores a la vacunación y posteriores a ella, y el rol de los refuerzos vacunales ^47^. Los resultados recientes de los estudios genómicos indican que la evolución reciente de *B. pertussis* está relacionada con la disminución de la inmunidad generada por las vacunas acelulares, en comparación con las vacunas de células completas, así como las variaciones genéticas de las cepas circulantes ^48^. Esto ha facilitado que los individuos afectados no sean solo los menores de edad, sino también, los adolescentes y los adultos.

## Tos ferina en Colombia

De manera similar a la historiografía europea, en Colombia tampoco hay mucha información sobre las epidemias de tos ferina. Pedro María Ibáñez (1859-1919), en sus *Memorias para la historia de la medicina en Santafé de Bogotá,* informó de una epidemia de coqueluche ocurrida en 1814 «que hizo numerosas víctimas» ^49^, pero no dio más detalles; esta es quizá la primera epidemia de tos ferina registrada en territorio colombiano.

En esa época, la ciudad estaba empezando a sufrir cambios importantes: se iniciaba la transición de ser una ciudad de gran extensión en relación con el número de habitantes y se aumentaba la concentración de individuos que habitaban en la misma vivienda. Se empezó a observar que la clase alta vivía en el segundo piso de las casas y los de clase baja, en las tiendas del primer piso, en condiciones de sanidad precaria ^50^. En este contexto, puede interpretarse que la enfermedad había llegado al país de forma poco frecuente y se había propagado entre los individuos susceptibles. El momento exacto del ingreso de la tos ferina a Colombia no se conoce; sin embargo, no debe olvidarse que años antes ya se habían descrito epidemias en Centroamérica.

En 1833, en la Facultad de Medicina de la Universidad Nacional, se publicó el *Tratamiento curativo del coqueluche* ^49^ y, en 1875, Liborio Zerda publicó en la *Revista Médica* un artículo en el que se invitaba a usar un nuevo tratamiento para el coqueluche. Partiendo de la existencia de un «parásito» causante de la enfermedad, el tratamiento consistía en la inhalación de ácido fénico, acompañado de antiespasmódicos como la belladona, adecuada alimentación y reposo ^51^. Estas publicaciones, sin duda, son evidencia de la importancia de la actualización para los médicos de la época. Dado que no hay informes de epidemias, como sí existen para otras enfermedades, es posible inferir el comportamiento endémico.

El médico Leopoldo Cajiao, en su tesis de grado titulada *Estudio general de la tos ferina y su nuevo tratamiento por la vacunación,* en 1916, hizo una revisión de la enfermedad y presentó 12 casos de menores de edad. Si bien no se describen epidemias específicas, es claro que era considerada una enfermedad de especial interés por «el carácter epidémico»:

«[...] En las grandes poblaciones llega a hacerse endémica, presentando a veces recrudecimientos, provocados por causas desconocidas, y se difunde entonces la enfermedad con una gran rapidez, amenazando la población infantil, sobre todo en las clases pobres y desvalidas, más expuestas por sus defectuosas condiciones de higiene al ataque de la infección […]» ^52^.

Esta descripción puede ayudar a comprender el comportamiento epidemiológico de la enfermedad, fundamentalmente como endemia, pero con episodios epidémicos de relativa poca frecuencia.

En 1922, Guillermo Márquez informó que desde finales de 1921 llegó a Bogotá una epidemia de tos ferina proveniente de la zonas norte y occidente del país. Indicó con claridad que los primeros casos fueron leves y se observaron después de finalizar una pandemia de gripe y una epidemia de rubéola de gran magnitud y poca duración. Hubo diferencias notorias en la gravedad de los casos según las diversas zonas, especialmente en Cajicá. Aunque hay descripciones clínicas, lastimosamente no hay muchos datos sobre su frecuencia, pero todo sugiere que el pico de incidencia se dio entre abril y julio de 1922; hubo 102 fallecidos entre enero y julio ^53^.

Cabe mencionar que Cajicá era un poblado pequeño dedicado a las labores agrícolas, que empezó su industrialización y la construcción de obras de servicio público, como la conexión con la electrificadora de Zipaquirá, a finales del siglo XIX. En 1899, en medio de un contexto político convulsionado en el municipio, apareció una empresa productora de sal ^54^ y se construyó la plaza, se pavimentaron calles y la carretera del norte, que actualmente corresponde a la autopista norte que comunica con Bogotá. Sin embargo, el evento histórico previo más relevante fue la pandemia de gripe española de 1918 a 1919 ^55^. Los cambios demográficos y, en general, los sociales pudieron haber jugado un rol importante en la génesis de la epidemia.

Durante la primera mitad del siglo XX, la tos ferina siguió ocupando uno de los primeros lugares de las causas de muerte en el país ^56^. Los registros nacionales de 1923 demuestran que las enfermedades respiratorias (incluyendo la tos ferina, las neumonías y el sarampión) predominaban entre las causas de muerte de niños pequeños ^57^.

Para tener una idea de la situación epidemiológica más específica de la tos ferina basta decir que:

«[...] en 1935 la mortalidad por tos ferina en el primer año de vida alcanzó 4,2 % en relación con todas las causas de defunción; sube dicha mortalidad en la edad preescolar a 6 %, y baja después de los 5 años» ^58^.

En 1948, el ministro de Higiene, Pedro Eliseo Cruz, informó que el Instituto Nacional de Higiene Samper-Martínez estaba renovando las cepas de *B. pertussis* para la producción de vacunas, incluyendo por primera vez cepas nacionales ^59^, lo que facilitó que, en 1952, la vacuna contra la tos ferina fuera incluida en el programa de vacunación infantil colombiano, junto con la viruela y la tuberculosis ^60^; el apoyo económico para la vacunación provenía de la OMS-OPS y de UNICEF ^61^. Si bien disminuyó la presentación de un buen número de casos con la vacunación, nunca se eliminó totalmente la infección, y se presentaron incrementos cíclicos de casos cada 2 a 4 años ^62^. Como referente, en 1983, en todo el país se registraron 5450 casos de tos ferina ^63^.

La vigilancia de la tos ferina fue de las primeras que se hicieron en Colombia; según el médico historiador Arturo Romero, los sistemas de vigilancia epidemiológica se fueron fortaleciendo a finales de la década de los 70, y se regularon con el Decreto 1562 de 1984 ^64^. De esa manera, se conoció mucho mejor la situación de la tos ferina y de otras enfermedades comunicables en el territorio nacional; además, la vigilancia fue la forma de seguir los avances del PAI en Colombia. En 1984, el PAI fue asumido como una cruzada nacional, lo cual permitió llegar a coberturas del 75 % en 1989 y del 87 % en 1990 ^43^, con lo que se logró disminuir la mortalidad infantil. La figura 1 muestra la disminución de los casos durante la segunda mitad del siglo XX ^65^.

**Figura 1:**
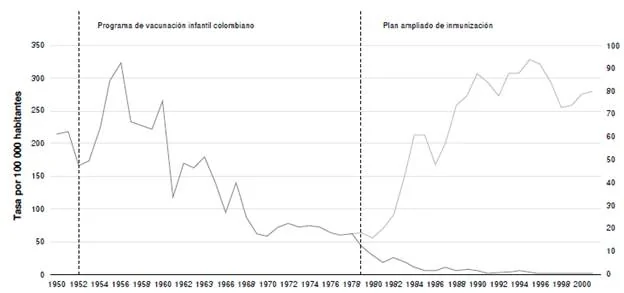
Incidencia de tos ferina en Colombia (1950-2001) y vacunación con DPT

* Datos estimados por Jiménez Peña 65 y del Ministerio de Salud y Protección Social

Un buen ejemplo de la situación nacional lo brinda el departamento de Antioquia que, en los tres primeros periodos epidemiológicos de 1982, recibió reportes de casos de tos ferina de 29 municipios, lo que motivó la búsqueda activa de casos que fueron reportados en el *Boletín Epidemiológico de Antioquia.* Así, en Barbosa, se detectaron 4 menores de edad con tos ferina, 2 de ellos sin haber sido vacunados ^66^; en Dabeiba, se confirmaron 2 casos de tos ferina, uno de ellos fallecido, y se identificaron 7 casos posibles en menores de edad. Entre los casos reportados en Dabeiba, únicamente 2 habían sido vacunados y ambos presentaban un esquema de vacunación incompleto ^67^.

En Valdivia, se reportaron 2 casos de tos ferina en menores de edad sin vacunar ^68^. De igual manera hubo informes epidemiológicos de casos en Concepción, Santa Bárbara, La Unión, La Estrella, Bello, Zaragoza, Caucasia y Santa Rosa de Osos. La falta de acceso a la vacunación en las zonas rurales fue el factor constante en estos casos. Pese a estos reportes de brotes, lo que puede verse es que las mejoras en la vigilancia permitieron identificar estos casos, verificar la necesidad de mejorar la vacunación en las zonas de difícil acceso y corroborar la efectividad de la vacunación ^69^.

El estudio de mortalidad evitable liderado por Rubén Darío Gómez, que pretendía evaluar las políticas sanitarias nacionales como consecuencia de la Ley 100 de 1993 -que organizó el nuevo sistema de salud-, reiteró la disminución de los casos de muerte por tos ferina en Colombia entre 1985 y 1993, y evidenció una tendencia al incremento entre 1994 y 2002 entre las mujeres ^70^. Años después, se empezó a señalar la reemergencia de la tos ferina y la necesidad de implementar medidas efectivas de salud pública, como la vacunación ^71^; se hablaba de que esta problemática era una tendencia global y, además, una consecuencia de la Ley 100.

El estudio que evidenció completamente esta problemática fue llevado a cabo por Doris Cardona *et al.,* quienes demostraron que la morbilidad y la mortalidad había aumentado entre el 2002 y el 2012, con incrementos más acentuados en el 2006, el 2009 y el 2012. Entre el 2005 y el 2012, hubo 1,88 casos por cada 1000 nacidos, y se pasó de 1,7 a 5,85 muertos por cada 1000 nacidos vivos en ese mismo periodo. Entre el 2002 y el 2012, hubo 173 fallecidos por tos ferina; el 97,1 % eran menores de un año. La frecuencia fue muy heterogénea entre los departamentos, pero se resalta que los brotes se presentaban principalmente en las instituciones educativas, en los que se identificaban adultos como casos índices ^72^.

Profundizando en el 2012, uno de los años con mayor frecuencia de la enfermedad después de la reemergencia, se destaca que hubo 3955 casos confirmados en todo el país, equivalente a 8,5 casos por 100 000 habitantes (61,7 y 255,6 casos por cada 100 000 menores de 5 años y menores de 1 año, respectivamente), y que el 61,2 % no tenía antecedentes de vacunación; además, ocurrieron 74 muertes, con una tasa de letalidad del 1,9 %. Se reportaron 46 brotes, principalmente en el departamento de Antioquia, en instituciones educativas. Además, en los brotes de Bogotá, Sucre, Cauca y Vaupés, hubo 5 fallecidos, 3 de ellos indígenas ^73^^,^^74^.

En Medellín, entre enero y septiembre del 2012, el incremento de casos fue objeto de estudio por varios autores. Se resalta la identificación del aumento de notificaciones diez veces superior al registrado en el 2011 para el mismo intervalo. La incidencia se estimó en 10,3 casos por cada 10 000 habitantes, siendo la población de 10 a 20 años la más afectada, seguida por los menores de 1 a 5 años. La letalidad calculada fue del 0,12 % ^75^.

Los brotes entre la población indígena resultan de especial interés dada la gran vulnerabilidad social de estos grupos étnicos. Entre estos hubo algunos brotes previos de tos ferina; por ejemplo, en el 2006 ocurrió un brote en el pueblo arhuaco de Jimain en Puerto Bello (Cesar) que afectó a 30 personas sin ocasionar muertes ^76^. En el 2009, ocurrió un brote en 322 individuos hitnü de Arauca; del total, se consideraron 54 casos probables y solo 2 pudieron confirmarse mediante pruebas de laboratorio, debido a dificultades con las muestras. No hubo fallecidos ^77^.

En el 2022, en la comunidad indígena kogui de Taminaka, Dibulla (La Guajira) en la Sierra Nevada de Santa Marta, se identificaron 62 casos de tos ferina, de los cuales 10 fallecieron; afectó principalmente a menores de 5 años y cerca del 94 % no tenía antecedentes de vacunación ^78^.

Debido al resurgimiento de la tos ferina en el mundo, en Colombia, la vigilancia se intensificó desde el 2010 mediante la búsqueda activa, y, para contrarrestar la elevada frecuencia entre menores de un año, se intensificó la vacunación. Como estrategia de prevención, desde el 2013 se empezó a vacunar a las mujeres con más de 26 semanas de gestación, con resultados efectivos rápidamente ^79^. Para contrarrestar esta reemergencia de la tos ferina, en el país se eligió la estrategia de la vacunación materna, como en otros países, y la mejora del diagnóstico entre los adultos mayores ^80^^,^^81^.

En Colombia, la serovigilancia en hospitales ha demostrado ser una herramienta útil para monitorear la inmunidad contra la tos ferina en las mujeres gestantes y en los recién nacidos. La experiencia de Antioquia (2015-2016) permitió estimar la prevalencia de anticuerpos maternos y en el cordón umbilical, y se destacó la vacunación de las mujeres gestantes como estrategia clave para proteger a los menores, aunque persisten retos de sostenibilidad y recursos ^82^.

Desde el 2017, con la llegada masiva de migrantes provenientes de Venezuela, se observó un incremento de los casos de tos ferina asociados con la falta de vacunación; en los años previos, se habían observado 1 o 2 casos y, en el 2018, se observaron 16 casos ^83^.

La pandemia de COVID-19 ocasionó una disminución de la cobertura de la vacunación infantil que, en el caso de la tos ferina, se vio afectada principalmente en la Amazonía, en los Llanos Orientales y en el Pacífico ^84^, regiones que históricamente han sido las de mayor vulnerabilidad social y que han dejado más niños susceptibles. Esto contribuyó a los brotes actuales, poniendo de relieve la importancia de mantener amplias coberturas de vacunación y vigilancia epidemiológica permanente. Un resumen de las epidemias y algunos brotes reportados se presentan en el cuadro 1.

**Cuadro 1:** Principales epidemias y brotes de tos ferina en Colombia

Año	Lugar	Descripción
1814	Santa Fe de Bogotá	Primera epidemia de tos ferina con «muchas víctimas»
1920	Sabana de Bogotá	Epidemia con 102 muertos que siguió a la pandemia de gripe y una epidemia de rubéola, que afectó principalmente a Cajicá.
1982	Antioquia	Brotes dispersos con pocos casos principalmente entre personas no vacunadas en varios municipios como: Barbosa, Dabeiba, Valdivia, Concepción, Santa Bárbara, La Unión, La Estrella, Bello, Zaragoza, Caucasia y Santa Rosa de Osos
2006	Puerto Bello, Cesar	Brote entre miembros del pueblo arhuaco; 30 casos, sin muertos
2009	Arauca	Brote entre individuos del pueblo hitnü; 54 casos, sin muertos
2012	Nacional	46 brotes, principalmente en Antioquia; también en Bogotá, Sucre, Cauca y Vaupés donde hubo 5 muertes, 3 de ellos indígenas
2012-2013	Antioquia	27 brotes, principalmente en establecimientos educativos de Medellín y Envigado
2022	Dibulla, La Guajira	Brote con 62 casos del pueblo kogui; 10 fallecidos

## Discusión

La revisión historiográfica aquí presentada demuestra que la tos ferina en Colombia ha tenido un comportamiento fundamentalmente endémico. Es probable que la llegada de la enfermedad al país haya sido en el siglo XVIII o a comienzos del XIX, dado que en 1814 ocurrió una epidemia importante en Bogotá y para ese tiempo ya había reportes de epidemias en otras regiones del continente americano. En su recorrido a la capital, ya fuera desde Perú, Venezuela o desde el Caribe, es posible que hayan ocurrido brotes de los que no hay informes escritos.

La otra epidemia importante ocurrió en 1922 en la sabana de Bogotá, principalmente en Cajicá. Sin embargo, la enfermedad siempre tuvo una gran frecuencia, siendo responsable de una proporción importante de las muertes entre los menores de edad.

Esta situación de elevada morbilidad y mortalidad entre los menores de edad y la escasa información sobre la tos ferina en Colombia resulta ser de gran interés. Primero, indica que las epidemias resultan ser más interesantes que las endemias para los médicos y quienes escriben la historia; hay un mayor interés de la sociedad por los eventos muy frecuentes y mediáticos ^85^. Segundo, y como hipótesis, los afectados eran los menores de edad, lo cual puede indicar la menor importancia que tenían en comparación con los adultos. No debe olvidarse *El niño y la vida familiar en el antiguo régimen,* escrito clásico del historiador francés Phillipe Ariès, que muestra la indiferencia que se tenía por los menores de edad, incluso cuando morían ^86^.

En Colombia, la importancia y atención a este grupo etario se empezó a manifestar como asunto de política pública solo a finales del siglo XIX ^87^. Quizá por ello la tos ferina no fue un asunto tan relevante, pese a la gran frecuencia endémica expresada en morbilidad y mortalidad entre los siglos XVII y XIX. Sin duda, este es un tema que requiere estudios más específicos.

Como ocurre con otras enfermedades infecciosas, cuando existen vacunas eficaces y campañas de vacunación efectivas, la historia muestra un antes y un después. El caso de la tos ferina es un caso exitoso de la vacunación en Colombia y en el mundo; no obstante, resulta necesario actualizar su estrategia para hacerla más efectiva. Para ello, se deberían incorporar las nuevas evidencias epidemiológicas que tienen en cuenta los cambios en los individuos afectados incluyendo adolescentes y adultos, y también los datos de los estudios genómicos que evidencian cambios evolutivos en las cepas de *B. pertussis,* con algún grado de resistencia a varias de las vacunas disponibles.

La incorporación de pruebas moleculares, proyectos como el *Latin American Pertussis Project* (LAPP) y la vacunación materna con dTpa, resaltan que la región avanza hacia estrategias más integrales ^46^. Como suele suceder, las poblaciones con mayor vulneración social son las prioritarias para ser atendidas.

La historia de la tos ferina en Colombia ha tenido diferentes momentos, dos periodos: antes de la vacuna y después de ella (figura 2). En el periodo de antes de la vacuna, el primer momento correspondió a la emergencia en el territorio, caracterizado por una ocurrencia creciente, aunque sin epidemias de gran magnitud. Luego hubo un largo periodo con variaciones cíclicas de incrementos cada 3 a 5 años, aproximadamente, que podría definirse como endémico de gran frecuencia. En este momento, se observaron epidemias francas, como las de Bogotá (1814) y Cajicá (1922), pero solo en condiciones muy específicas que parecen relacionarse con los cambios sociales y las transformaciones ambientales urbanas. Con la aparición de la vacunación se observó un primer momento de rápido descenso, que permitió identificar brotes aislados en poblaciones con gran vulnerabilidad social al mejorar la vigilancia. El segundo momento, que incluye el momento actual, se caracteriza por la importación de casos.

**Figura 2:**
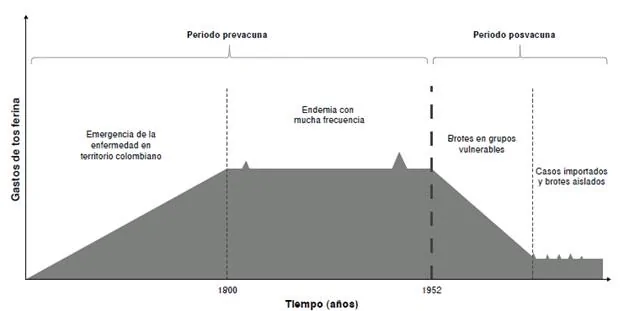
Periodos y momentos característicos de la historia de la tos ferina en Colombia

## Conclusiones

La tos ferina fue una enfermedad emergente en América, que llegó con los europeos y ocasionó elevada mortalidad en las poblaciones indígenas sin inmunidad. Su propagación por el territorio colombiano fue más lenta que la de otras enfermedades como la viruela, la gripe y el sarampión. Las evidencias sugieren que al inicio de la Conquista la tos ferina encontró, principalmente entre los indígenas, una población que le permitía mantenerse como endemia.

La natalidad debió jugar un rol preponderante en el mantenimiento de la tos ferina, por lo que los lugares donde hubo mayor número de nacimientos en condiciones no higiénicas debieron ser los que debieron haber mantenido la enfermedad y, en los casos señalados, facilitaron las epidemias. La vacunación resultó ser la intervención más apropiada para prevenir la enfermedad y la muerte asociada.

Actualmente, la tos ferina es un desafío para la salud poblacional colombiana, que requiere atención para no retroceder en los éxitos ya alcanzados. Los brotes de los años recientes muestran la punta del iceberg de una sociedad inequitativa, donde los más vulnerables siguen siendo los que mayor riesgo tienen de enfermar o morir de tos ferina.
